# Feasibility study of three-dimensional printing knee model using the ultra-low-dose CT scan for preoperative planning and simulated surgery

**DOI:** 10.1186/s13244-022-01291-8

**Published:** 2022-09-24

**Authors:** Meng Zhang, Ming Lei, Jie Zhang, Hongyi Li, Fenghuan Lin, Yanxia Chen, Jun Chen, Mengqiang Xiao

**Affiliations:** 1 Zhuhai Hospital, Guangdong Provincial Hospital of Traditional Chinese Medicine, 53 Jingle Road, Zhuhai City, Guangdong Province China; 2 Zhuhai People’s Hospital, Zhuhai City, Guangdong Province China

**Keywords:** Three-dimensional printing, Knee, Ultra-low-dose

## Abstract

**Objective:**

To explore the feasibility of the three-dimensional printing (3DP) knee model using the ultra-low-dose computed tomography (CT) scan for preoperative planning and simulated surgery.

**Methods:**

Thirty-six patients were divided into the standard-dose protocol group (A) and ultra-low-dose protocol group (B). The anteroposterior diameter, left and right diameter of femur, anteroposterior diameter of tibial plateau (APTP), left and right diameter, distance from the intercondylar ridge to tibial tuberosity, lower femur angle, and upper tibial angle were measured on CT images. On the 3D printed knee joint model, Vernier calipers were used to measure: anteroposterior diameter, left and right diameter of the internal and external condyles of femur; left and right diameters, anteroposterior diameters of tibial plateau; upper and lower meridian, left and right diameters of patella.

**Results:**

With group A as reference, the effective radiation dose in group B was significantly reduced to 97.0% (36.4 ± 3.7 uSv and 1.1 ± 0.2 uSv, respectively). There was no difference in objective parameters for 3DP model (*p* = 0.31–0.84). None of the quantitative parameters of image quality showed significant difference (*p* = 0.11–0.96). Despite lower score of image quality and 3DP model in group B (3.0 ± 0.0 vs. 2.1 ± 0.2, 2.9 ± 0.3 vs. 2.2 ± 0.4; *p* < 0.05), the diagnostic performance was consistent in the two groups (all scores ≥ 2). Image quality and 3DP printed models were highly consistent (*k* = 0.97).

**Conclusions:**

Ultra-low-dose protocol reduces the radiation dose while maintaining the image quality of knee. It meets the requirement for 3DP model, internal fixation model selection, and simulated surgery.

## Key points

- ULD-CT qualified the requirements for 3DP model, internal fixation model, and simulated surgery.
- Ultra-low-dose CT schemes for the knee disorders are lower than DR.
- Ultra-low-dose protocol for knee imaging effectively reduces the dose of radiation.

## Background

During the period 1990–2015, the global life expectancy increased from 63.5 years in 1990 to 71.8 years in 2015 [1, 2]. Owing to the progressive population aging, the number of people with knee disorders is gradually increasing. Of these, osteoarthritis is a common cause of knee dysfunction, which imposes a significant burden on the affected individuals and the society at large. Knee arthroplasty, including unicompartmental knee arthroplasty (UKA) and total knee arthroplasty (TKA), is indicated for treatment of end-stage knee osteoarthritis. According to estimates, the number of knee arthroplasties performed in US in individuals aged ≥ 65 years increased by 162% in the period between 1991 and 2010, and up to 600% increase in rates is projected by the year 2030 [3].

Recent years have witnessed widespread use of three-dimensional printing (3DP) technology in various fields [4–8]. In the field of orthopedics, this technique is mainly used for preoperative morphological design, intraoperative tissue repair, and reconstruction of large bone defects. Use of 3DP products during surgery has enabled resolution of complex problems. 3DP models based on CT images provide the surgeon with a clearer anatomical view in full 3D which facilitates preoperative digital planning of osteotomy. This technique has been shown to improve preoperative planning, intraoperative precision, and to reduce postoperative complications [9]. This approach has been shown to be particularly beneficial in improving the accuracy of intraoperative osteotomy, the mechanical strength and stability of the prosthesis, and postoperative stability of the knee joint [10].

For CT imaging, the traditional focus has been on reducing tube voltage and tube current. However, recent research has focused on ways to improve image quality using new algorithms. The reduction of voltage and current directly reduces the dose of radiation exposure. However, such a reduction inevitably increases the noise and affects the accuracy of diagnosis. In recent years, with the development of follow-up computer technology, iterative algorithms have been applied in clinical settings to improve the image quality, especially in low-radiation CT scanning. Using this algorithm, the image quality can be improved by the model, and the clinical diagnosis needs can be met [11–15].

Increasing use of CT scan has aroused concerns about radiation exposure. Although CT imaging studies account for merely 11% of radiologic procedures, these account for approximately 70% of the total effective dose [16]. The purpose of this study was to explore the effect of use of ultra-low-dose CT scanning for developing 3DP knee model, selection of internal fixation model, and simulated surgery.

## Methods

This study was approved by the institutional research ethics committee of the Guangdong Provincial Hospital of Traditional Chinese Medicine (BF2019-030-01). Written informed consent of patients was obtained. Between February 2019 and August 2020, 36 patients (21 men and 15 women) were treated at the Zhuhai Provincial Hospital of Guangdong Hospital of the Traditional Chinese Medicine Hospital. An average of two knee joint scans were performed per patient. These included 20 patients who were undergoing conservative treatment of knee fractures (CT re-examination was required for non-operated fracture patients within 2 weeks to evaluate any increase in the degree of fracture displacement necessitating surgery), 2 patients with a history of repeat injury after the first CT scan, and 14 patients who underwent cruciate ligament reconstruction (one CT scan each before and after surgery). The first CT scan was performed using standard-dose protocol, while the second CT scan used ultra-low-dose protocol; the mean interval between the two scans was 8.0 ± 2.1 days. The mean age of patients was 43.8 ± 15.3 years (range 19–82). Patients aged ≥ 18 years were included. Patients aged < 18 years and pregnant women were excluded.

### Scan method and technique details

All CT scans were performed using Canon 320-slice dynamic CT (Canon Aquilion One, Japan). According to the previous research results, the CT scan parameters were as follows: FOV: 20 mm; reconstruction layer thickness: 0.5 mm; spacing: 0.5 mm. In conventional CT scan (according to the textbook and parameters of Chinese medical imaging technology CT imaging technology jointly developed by Canon Engineers), CT scan parameters were as follows: tube voltage 120 kV; tube current 100 mAs. For ultra-low-dose CT, the scanning parameters were as follows: tube voltage 80 kV, tube current 11 mAs. The scanning conditions for each group are shown in Table 1.

**Table 1:** CT parameters of each group

Dose group	Tube voltage (kV)	Tube current (mAs)	D-FOV (mm)	Rotation time (s)	Thickness (mm)	Interval (mm)	Scan length (mm)	AIRD3D
Standard	120	100	180	0.75	0.5	0.5	140	Standard EU10
Ultra-low	80	11	180	0.75	0.5	0.5	140	Standard EU10

### 3D printer

The original CT data were reconstructed iteratively to obtain the data package. The layer thickness and layer spacing of the data package were 0.5 mm. The data package was imported in the reconstruction software Mimics research 21.0 and the slice software (Simplify3D V4.0). According to the reconstruction data, the skeleton model using the melting deposition method was printed using Tianwei coliDo 3.0 3D printer. The printing material was diameter 1.75 mm white polylactic acid.

### Radiation dose

Computed Tomography Dose Index volume (CTDIvol) (mGy) and Dose length product (DLP) (mGy * cm) were automatically indicated by the scanner software for all CT-protocols. In order to obtain the effective dose (ED = DLP * *k*), DLP for each patient was multiplied by *K* (a conversion coefficient). For the extremity scans, *k* = 0.0002 has been used for extremity scans [17].

### Image evaluation

#### Objective indicators

Vernier calipers were used to measure the following indicators on the 3D printed model of knee joint: anterior and posterior diameter and the left and right diameter of the internal and external condyles of the femur (APDLF: Anterior and posterior diameter of lateral femoral condyle; APDFC: Anterior and posterior diameter of the femoral medial condyle; LRDMLFC: Left and right diameters of medial and lateral femoral condyles). Left and right diameters and anteroposterior diameters of tibial plateau (LRTP: Left and right diameters of tibial plateau; APTP: anterior and posterior diameters of tibial plateau). The upper and lower meridian and left and right diameters of the patella (ULP: Upper and lower meridian of the patella, LRDP: Left and right diameters of the patella). The anteroposterior diameter, left and right diameter of the femur, the anteroposterior diameter of the tibial plateau, the left and right diameter, the distance from the intercondylar ridge to the tibial tuberosity, the lower femur angle, and the upper tibial angle were measured on the CT images of the knee joint (Canon Workstation, Canon Aquilion One, Japan). The above parameters play an important role in the selection of the internal fixation model. Three measurements were taken, and the average value was used for analysis.

#### Subjective indicators

Two senior radiologists with at least 7 years of experience in diagnostic radiology participated in this evaluation. They had not participated in the clinical optimization of the previous hybrid system and were blinded to the patient’s clinical information. Both radiologists independently reviewed the collection of images in a random order using a dedicated workstation. Individual optimization was also allowed for the window, including the subjective image quality and detectability of structures. Referring to scholar CT scan for image quality assessment [18, 19], the image quality of anatomical structures and image noise was scored on a 3-point scale defined as follows: Score 1–Distortion of spatial resolution or contrast resolution or impossible edge definition due to high image noise; image quality is poor and unevaluable; image noise is very high with reading restrictions, and a diagnosis is difficult or even impossible. Score 2–Adequate image quality, affected by image noise, or some spatial resolution or contrast resolution distortion; but edge definitions are fully present; images can be read without distraction, enough image noise is present, not distracting reading, but still allowing a correct diagnosis. Score 3–Good image quality, with only minimal image noise, or minimal spatial resolution or contrast resolution distortion; image noise has no effect on fracture diagnosis.

Two senior orthopedic attending doctors used the 3-point evaluation method to assess the definition of the 3D printed model and the guidance of the operation. 3 points: the 3D printed model has a smooth surface, which has no adverse influence on the design of the operation plan, preoperative operational practice, and intraoperative auxiliary operation [20]; 2 points: slightly rough surface of 3DP model, which has no adverse influence on the design of the surgical scheme, preoperative simulation of the operation, and the intraoperative auxiliary operation; 1 point: rough surface of the 3DP model, which has a great influence on the design of the surgical scheme, preoperative simulation of the operation, and intraoperative auxiliary operation [20].

Thirty-six cases of knee joint patients with standard-dose and ultra-low-dose data packets were sent to AK Medical Group, and the mincs system was used to simulate total knee replacement to select surgical methods and internal fixation models.

### Statistical analysis

Statistical analyses were conducted using SPSS software, version 27.0 (IBM Corp, Armonk, NY, USA). Data normality was assessed using the *T* test. Differences with respect to CTDIvol, ED, and objective indicators were assessed using the *t* test. Image quality scores and 3DP model scores were analyzed using the rank sum test. The Kappa test was used to estimate the consistency between the evaluation of 3D printing model by 2 doctors (Kappa value < 0.04: slightly consistent; 0.41–0.60: moderately consistent; 0.61–0.80: highly consistent; 0.81–1.00: almost completely consistent). *p* values < 0.05 were considered indicative of statistically significant difference.

## Results

**Fig. 1:**
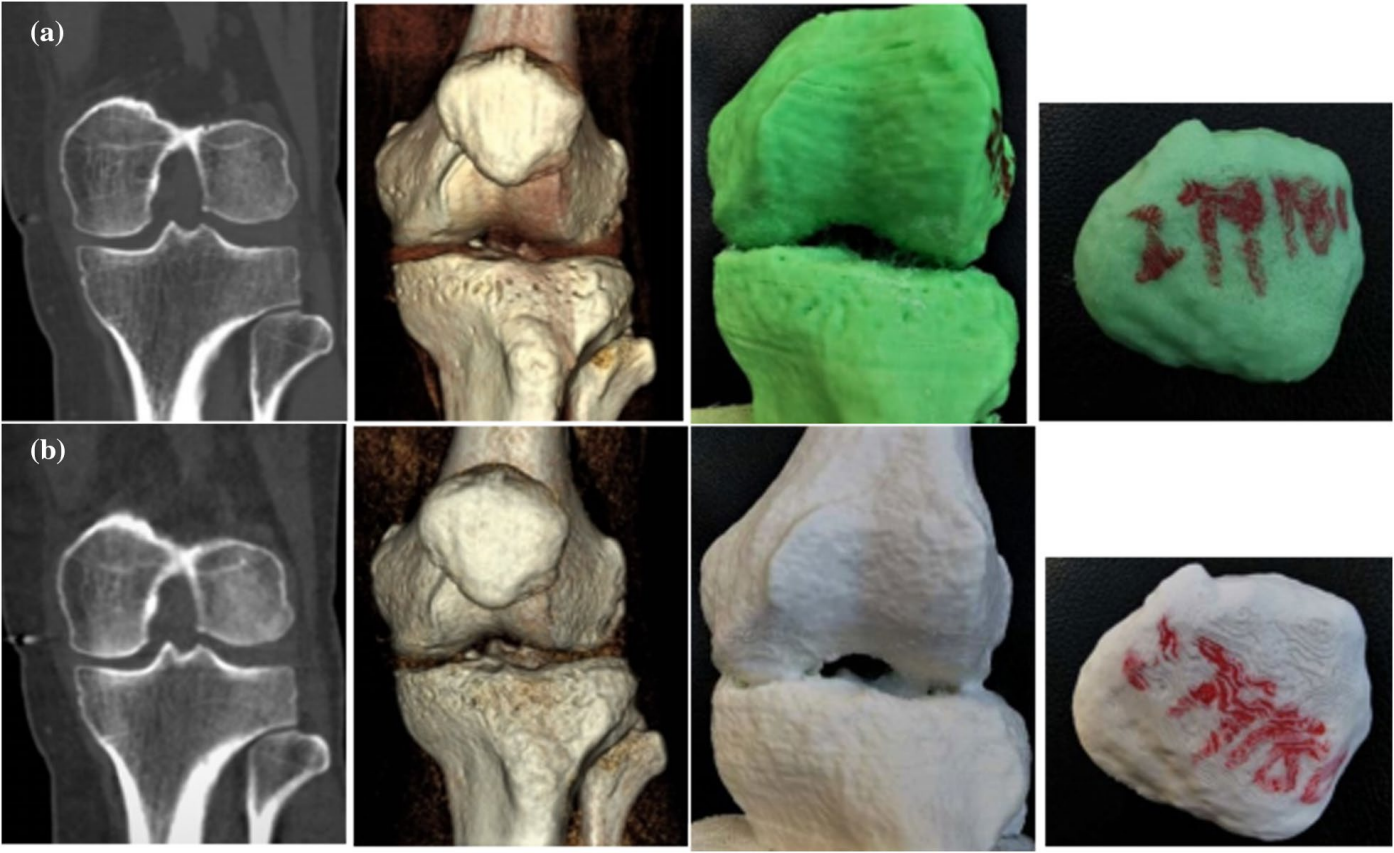
Representative case study 1. A 62-year-old man with a suspected intercondylar ridge fracture underwent two scans (at the first visit and 7 days later). Standard-dose (**a**) and ultra-low-dose (**b**) protocols were used to generate three-dimensional printing models based on 3D CT data. Image quality and 3D printing model based on the former is better than the latter (3 vs. 2 points). The 3DP model is appropriate for preoperative planning

**Fig. 2:**
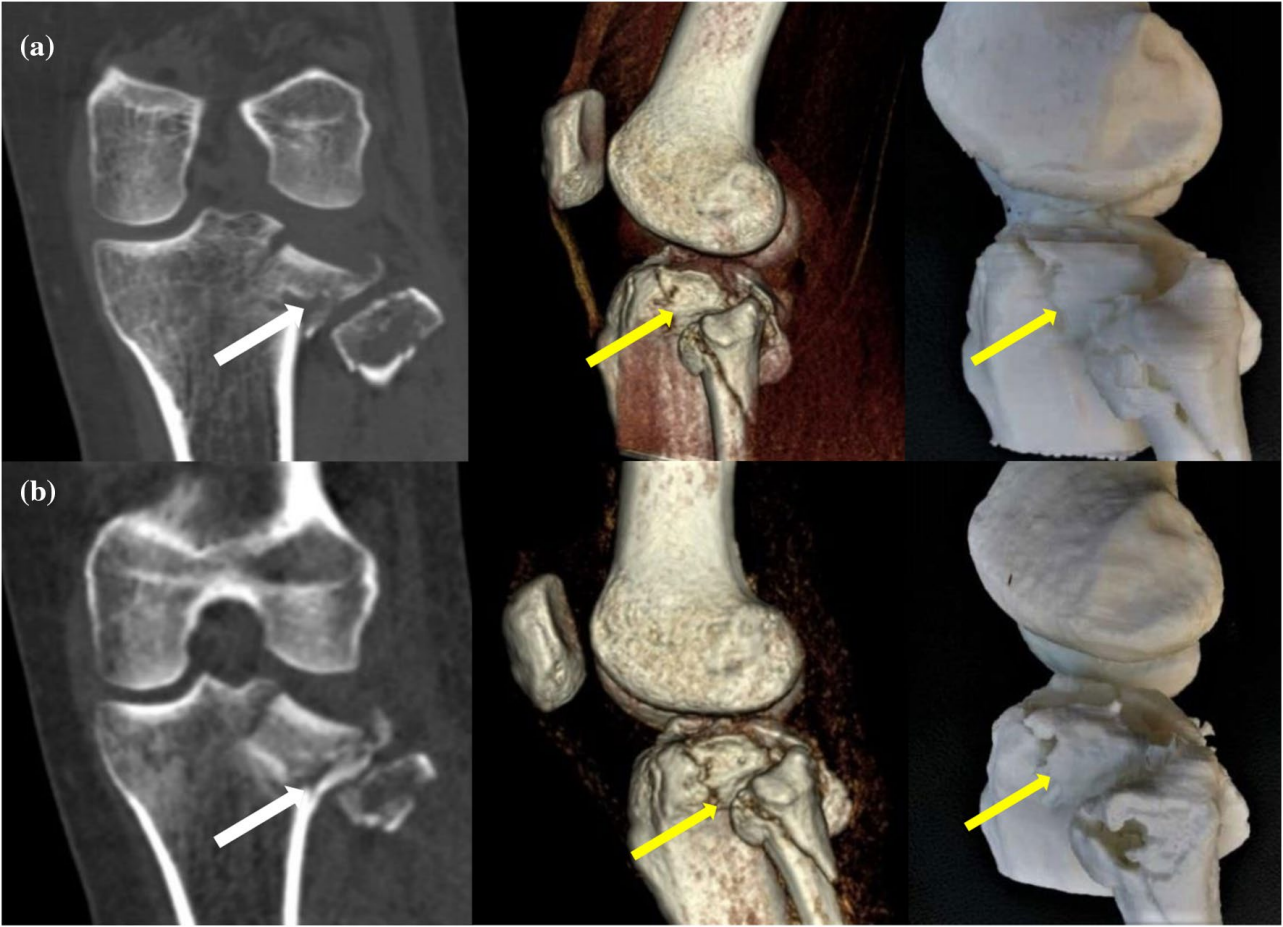
Representative case study 2. A 36-year-old woman with comminuted fracture of tibial plateau and fibula underwent two scans (at the first visit and 8 days later). The standard-dose (**a**) and ultra-low-dose (**b**) protocols were used to generate three-dimensional printing models from 3D CT data. The direction of the image quality (white arrows) and locations of the fragments (yellow arrows) are identifiable with both scans, meeting the needs of clinical diagnosis and treatment. The 3DP score was 3 points. The surface of the bone models is regular and flat, which is suitable for preoperative evaluation

**Fig. 3:**
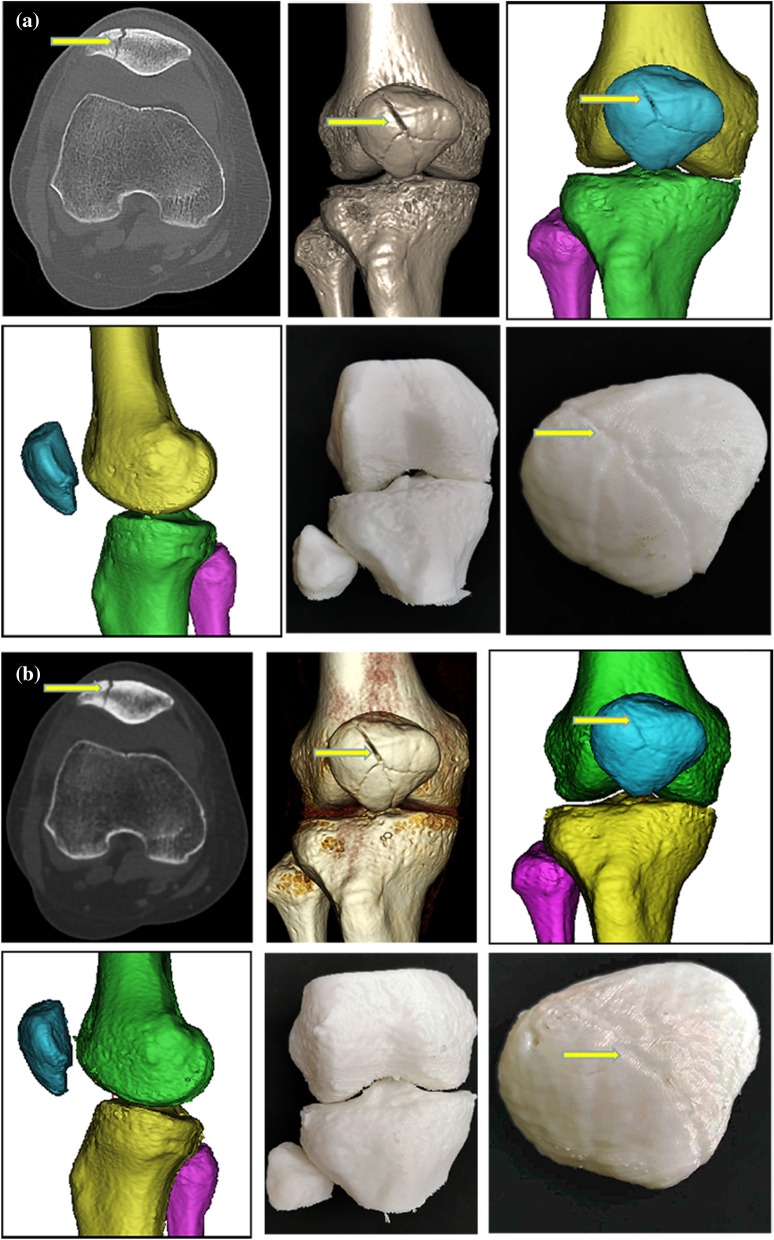
Representative case 3. A 47-year-old woman with undisplaced patella fracture underwent two scans (at the first visit and 10 days later). The standard-dose (**a**) and ultra-low-dose (**b**) protocols were used to generate 3-D printing models from 3D CT data. **a**-1 and **b**-1 CT axial images with quality score of 3 points in **a** and 2 points in **b**. The fracture lines (yellow arrows) are identifiable in both scans, meeting the needs for clinical diagnosis and treatment. **a**-2 and **b**-2 Maximum intensity projection images. **a**-3–4 and **b**-3–4 3D reconstruction of Mimics research medicine images. **a**-5–6 and **b**-5–6 3DP images with score of 3 points in **a** and 2 points in **b**. The surface of the bone models is regular and flat, which is suitable for preoperative evaluation

Data pertaining to CTDIvol, ED, SD, objective evaluation of image quality and 3D printed model in the two groups are summarized in Table 2. The average CTDIvol in the standard and ultra-low-dose group was 11.6 ± 0.7 mGy and 0.3 ± 0.05 mGy, respectively, while the ED was 36.4 ± 3.7 uSv and 1.1 ± 0.2 uSv, respectively. The CTDIvol and ED in the ultra-low-dose group was 2.3% and 2.9% lesser than that in the standard-dose group (Table 2). The subjective scores of image quality and 3D printed model in the standard group were significantly better than those in the ultra-low-dose group (*p* < 0.05). Both groups qualified the requirement of accurate clinical diagnosis (Figs. 1, 2, 3a, b).

**Table 2:** Subjective evaluation of 3D printed model and Image quality and parameters of each group

Dose group	3D printed model score	Image quality score	CTDI_vol_ (mGy)	DLP (mGy * cm)	ED (uSv)
Standard	3.0 ± 0.2	3.0 ± 0.0	11.6 ± 0.7	182.1 ± 18.5	36.4 ± 3.7
Ultra-low	2.1 ± 0.3	2.1 ± 0.2	0.34 ± 0.05	5.3 ± 0.5	1.1 ± 0.2
Sig	0.00	0.00	0.00	0.00	0.00

**Fig. 4:**
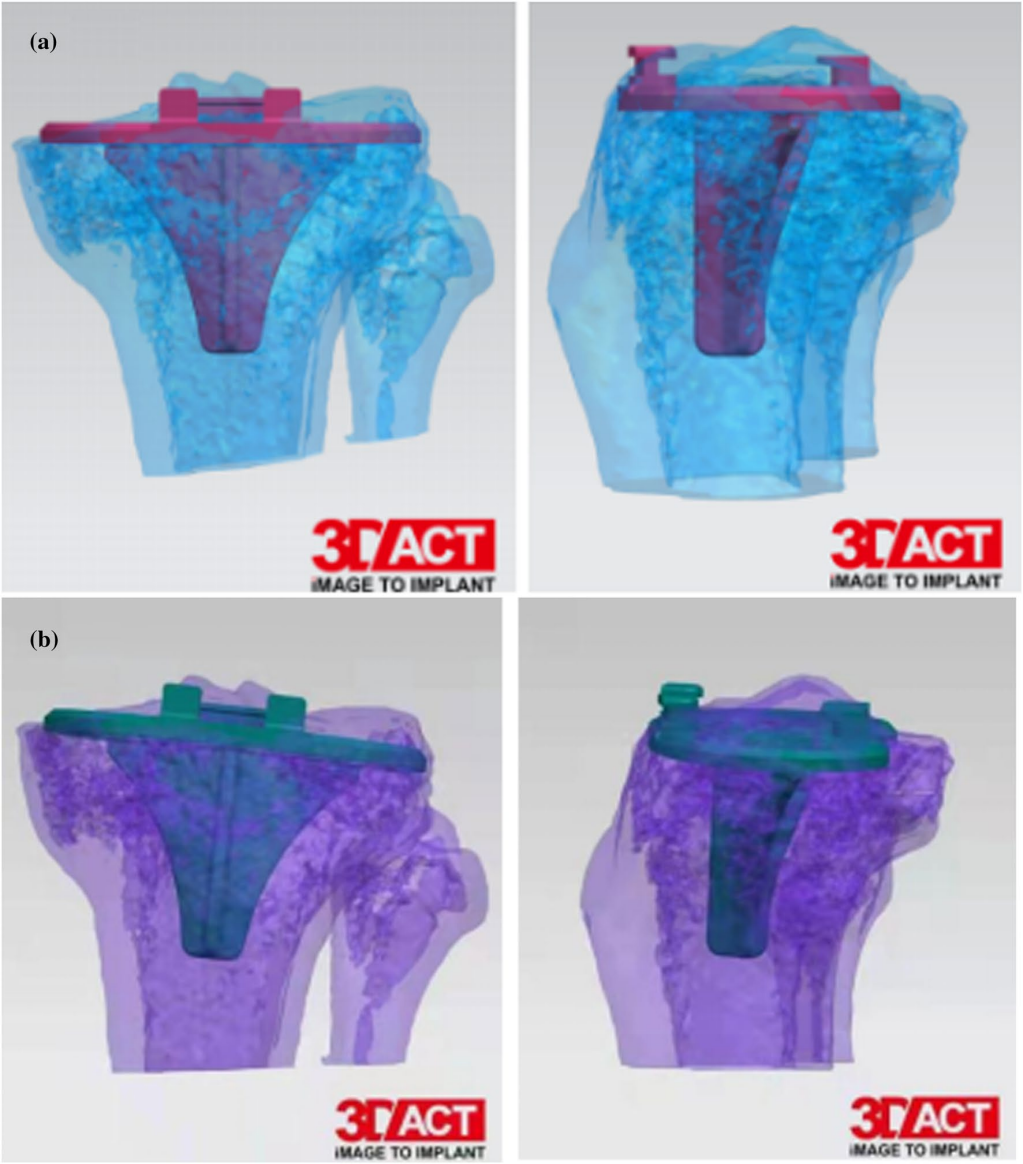
Representative case study 4. A 62-year-old man. **a** Simulated operation and internal fixation based on standard-dose CT scan; **b** simulated operation and internal fixation based on ultra-low-dose CT scan. The internal fixation models of **a** and **b** are identical, and the operation method is the same

There were significant differences between the standard-dose and ultra-low-dose 3D models with respect to Vernier caliper measurements and CT image measurements (Tables 3, 4). A high consistency was observed between the image quality scores awarded by the two radiologists (*k* = 0.97). The reliability score of the 3D printed model by the two orthopedicians was 3 points, and the results were highly consistent (*k* = 0.97). The simulated knee surgery and internal fixation models based on standard-dose and ultra-low-dose CT images were not different in the entire cohort (Fig. 4).

**Table 3:** Measurement parameters of 3D printed model

Dose group	APDLF (mm)	LRDMLFC (mm)	APDFC (mm)	ULP (mm)	LRDP (mm)	LRTP (mm)	APTP (mm)
Standard	6.3 ± 0.5	8.0 ± 0.6	6.4 ± 0.5	4.3 ± 0.9	4.6 ± 0.8	7.4 ± 0.5	5.2 ± 0.5
Ultra-low	6.4 ± 0.5	8.0 ± 0.6	6.5 ± 0.5	4.2 ± 0.9	4.6 ± 0.5	7.4 ± 0.5	5.2 ± 0.5
Sig	0.47	0.80	0.31	0.76	0.84	0.76	0.80

**Table 4:** Measurement parameters of CT imaging

Dose group	LRDMLFC (mm)	APF (mm)	LRTP (mm)	APTP (mm)	DICTT (mm)	Upper tibial angle (°)	Inferior femoral angle (°)
Standard	7.9 ± 0.6	6.5 ± 0.6	7.4 ± 0.2	5.3 ± 0.5	5.6 ± 0.8	87.7 ± 5.809	86.3 ± 5.5
Ultra-low	8.1 ± 0.8	6.6 ± 0.5	7.6 ± 0.5	5.4 ± 0.4	5.6 ± 0.8	87.3 ± 6.1	86.2 ± 5.3
Sig	0.37	0.35	0.11	0.16	0.80	0.94	0.96

## Discussion

To the best of our knowledge, there is a paucity of studies that have assessed the use of ULD-CT scan for 3DP of knee joint. The radiation exposure in the ULD-CT group was < 1.1 uSv (33 times lower than chest X-ray and 2 times lower than hands and feet X-ray) [21, 22]. Although ultra-low-dose CT reduces the quality of 3D printed models and CT images, it can meet clinical needs (image score ≥ 2 points). No significant difference of the parameters was observed in the 3D printed model. It accurately simulated the postoperative methods and internal fixation models, and surgery. There was no difference with respect to the method and internal fixation model selection.

According to previous studies, the CT data obtained should meet the following requirements: recommended scanning layer thickness and layer spacing ≤ 1 mm [6]. At present, only 3D printing model is closely related to CT scan thickness, while CT scan thickness and interval are not related to CT scan radiation dose. To the best of our knowledge, the influence of ultra-low-dose CT scan on orthopedic 3DP knee model is not well characterized. Reduction of the radiation dose increases the noise to a certain extent, which reduces the smoothness of the edges of the reconstruction model and introduces artifacts; such model may not truly reflect the bone morphology. In this study, the same patient was scanned by CT twice, the first was conventional CT scan, and the second was ultra-low-dose CT scan. The thickness and spacing of the image layers were the same (0.5 mm), and the 3D printed model was printed. The objective and subjective differences of the two 3D-printed knee joint models were evaluated. This study showed no difference between standard-dose and ultra-low-dose CT scans with respect to the size of femur, tibia, and patella; in addition, there was no significant difference in the size of the 3D model as measured by the Vernier caliper. The surface of the model was relatively smooth, which qualified the requirements of clinical 3DP. The image quality of ultra-low-dose CT scan can meet the needs of clinical diagnosis of degenerative changes and fractures.

Accurate bone resection in TKA requires correct delineation of the contours of the knee joint and soft tissue. Due to the complexity of bone and soft tissue resection and reconstruction of TKA, surgeons typically use two-dimensional CT and magnetic resonance images for preoperative planning. Surgical planning for TKA is difficult without the aid of a computer because of the complex regional anatomy. In recent years, use of the 3D guide template has enabled precise resection of bone and accurate implantation of the knee joint prosthesis, which reduces the time of radiation and operation, and avoids neurovascular injury caused by malpositioned screws [23–26]. This study shows that the ultra-low-dose 3DP model can meet the needs of clinical surgery and internal fixation model selection.

Some limitations of this study should be acknowledged. First, we only evaluated the choice of simulated surgery and internal fixation model using ultra-low-dose CT. It is not clear whether use of ultra-low-dose CT scan for this purpose affects the surgical outcomes. Secondly, we focused on the quality of CT images and 3DP models, but we did not assess the diagnostic accuracy of the CT images and the 3DP models by comparing with the reference standard. Thirdly, we compared the images and models quality on a particular vendor. However, scanners from other vendors may offer higher image quality with lower radiation dose using the same scan parameters settings with different materials of tube or detector and iterative reconstruction algorithm.

## Conclusions

The present study demonstrates the feasibility of 3DP knee model using the ULD-CT scan for preoperative planning and simulated UKA and TKA. Ultra-low-dose protocol for knee imaging effectively reduces the dose of radiation, while maintaining the image quality. ULD-CT qualified the requirements for generation of 3DP model, selection of internal fixation model, and simulated surgery. Therefore, this method is a viable option for use in clinical settings.

## Data Availability

The datasets used or analyzed during the current study are available from the corresponding author on reasonable request.

## Abbreviations

ADF: Anteroposterior diameter of femur
APDFC: Anterior and posterior diameter of the femoral medial condyle
APDLF: Anterior and posterior diameter of lateral femoral condyle
APTP: Anteroposterior diameters of tibial plateau
DICTT: Distance from intercondylar crest to tibial tuberosity
LRDMLFC: Left and right diameters of medial and lateral femoral condyles
LRDP: Left and right diameters of the patella
LRTP: Left and right diameters of tibial plateau
TKA: Total knee arthroplasty
UKA: Unicompartmental knee arthroplasty
ULP: Upper and lower meridian of the patella
